# Association of LncRNA PCBP1-AS1 with cancer occurrence and development: A review

**DOI:** 10.1097/MD.0000000000035631

**Published:** 2023-10-27

**Authors:** Yanping Wu, Jie Mou, Yuling Liu, Wenfei Zheng

**Affiliations:** a Department of Gynecology and Obstetrics, The First college of China Medical Science, China Three Gorges University/Yichang Central People’s Hospital, Yichang, Hubei, China.

**Keywords:** cancer, long noncoding RNA, PCBP1-AS1, regulation, therapeutic target

## Abstract

Long-stranded noncoding RNAs (LncRNAs) are noncoding RNAs >200 nucleotides in length. Polycytidine binding protein 1 antisense LncRNA is abbreviated as LncRNA polycytosine binding protein 1 antisense1 (PCBP1-AS1). Since studies in recent years have revealed the importance of PCBP1-AS1 in human genetic analysis, it is an important member of the LncRNA family. Genetically engineered group analysis of PCBP1-AS1 regulates the progression of cancer in biology. Therefore, it may be an important RNA in the regulation of human cancer. This article summarizes the molecular mechanism and clinical role of PCBP1-AS1 in various tumor types. Taking “PCBP1-AS1” and “cancer” as keywords, this paper analyzed the relationship between PCBP1-AS1 and various tumors by searching PubMed and Geen Medical, and summarized the related regulatory mechanism of PCBP1-AS1. PCBP1-AS1 is a valuable tumor-associated LncRNA that plays different biological roles in different cancers. Overall, it can both promote and inhibit the development of cancer. For example, abnormally high expression in castration-resitant prostate cancer, hepatocellular carcinoma, cervical cancer, glioma, and colorectal cancer promotes the proliferation and progression of these cancers; in contrast, PCBP1-AS1 inhibits cancer proliferation, metastasis, invasion, and recurrence when highly expressed in vulvar squamous cell carcinoma, Hodgkin lymphoma, and lung adenocarcinoma. PCBP1-AS1 regulates the development of multiple tumors, and the specific mechanism needs to be further investigated, which may become a new tumor marker and potential therapeutic target.

## 1. Introduction

Understanding the molecular basis of disease and designing reasonable molecular therapies have been the holy grail of human cancer management, and an important way to achieve a possible cure is gene therapy.^[1]^ Long noncoding RNAs (LncRNA) are RNAs with a transcription length of more than 200 nucleotide and lack protein-coding capabilities^[2]^ (Protein-coding genes comprise approximately 20% of the entire human genome, and 80% of the human genome is transcribed into RNA, but these RNA transcripts lack protein-coding capacity and are therefore noncoding^[3]^). Although LncRNA do not encode proteins, some aspects of LncRNA biology are similar to messenger RNA (mRNA), and most LncRNAs can also be transcribed by RNA polymerase II (Pol II).^[4]^ Although the expression levels of LncRNAs are usually lower than those of mRNAs, they show stronger tissue-specific expression patterns, indicating that LncRNAs still play very broad roles in the life activities of organisms, such as gene modification, RNA shearing, editing, microRNA (miRNA) regulation and protein folding.^[5,6]^ It is well known that a variety of lnc RNA are involved in regulating the energy metabolism of cancer,^[7]^ such as LUCAT1,^[8]^ DUXAP10,^[9]^ GAS5,^[10]^ TTN-AS1,^[11]^ etc.

Cancer is a worldwide problem, which is characterized by infinite proliferation, invasive metastasis and the difficulty of early stage, and it has not been overcome yet. Therefore, early detection of some tumor markers can effectively improve the chance of malignant tumor diagnosis. According to studies, polycytosine binding protein 1 antisense1 (PCBP1-AS1) plays an important role in the progression of many cancers, including destructive resistant prostate cancer, hepatocellular carcinoma, lung adenocarcinoma, cervical cancer (CC), vulvar squamous cell carcinoma, Hodgkin lymphoma, glioma, colorectal cancer, and oral squamous carcinoma. It is currently believed that LncRNAPCBP1-AS1 is widely involved in the proliferation, invasion, metastasis, drug resistance, and recurrence of malignant cells, but the regulatory mechanisms differ in different cancers.

In recent years, the role and potential mechanism of PCBP1-AS1 in human cancer has been a relatively new topic. A number of studies have emerged to confirm the correlation between PCBP1-AS1 as a tumor marker and cancer, but it seems that more research results are needed to support the clinical use of some hard-to-treat cancers at this time. This paper presents a literature review of the association between noncoding RNA PCBP1-AS1 and cancer based on the research literature that has emerged so far, and attempts to illustrate the regulatory mechanisms of PCBP1-AS1 in different cancers.

## 2. Performance of LncRNA PCBP1-AS1 in different cancer

### 2.1. Castration-resistant prostate cancer

Prostate cancer occurs in the male prostate gland as a result of abnormal and uncontrolled growth of prostate alveolar cells. Castration-resistant prostate cancer (CRPC) is the final result of treatment for most other prostate cancers.^[12]^ The latest research shows that the incidence and death of prostate cancer in Chinese men are still rising sharply.^[13]^ Although androgen deprivation therapy is currently one of the most effective remedies, the resistance to drug use means that androgen deprivation therapy therapy fails.^[14]^ Therapies that completely inhibit tumor growth through drugs face enormous challenges. So, finding molecular targets to inhibit the growth of prostate cancer cells is of great significance for the treatment of prostate cancer.

The study by Zhang et al^[15]^ found a remarkable increase in PCBP1-AS1 in CRPC compared to adjacent normal tissues. The development of destructive resistant prostate cancer is strongly correlated with aberrant activation of androgen receptor (AR) signaling pathways such as AR-V7. PCBP1-AS1 is involved in the AR ubiquitin-proteasome degradation process, which in turn makes CRPC more resistant to drugs. In addition, studies have shown that PCBP1-AS1 and AR protein have high binding potential, and the expression levels of them are positively correlated. PCBP1-AS1 high expression promotes the growth and drug resistance of destructive resistant prostate cancer. Conversely targeting PCBP1-AS1 and AR signaling pathway inhibit tumor progression. However, the study of the exact molecular mechanism is still in its early stage, and an in-depth study of the underlying mechanism may provide new ideas for the treatment of prostate cancer.

### 2.2. Hepatocellular carcinoma

Hepatocellular carcinoma is referred to as hepatocellular carcinoma (HCC), which is the main histological subtype of liver cancer and is one of the most common cancers in the world.^[16]^ It is a major cause of death in cancers.^[17]^ Metastatic liver cancer is the major killer, and the refractoriness of metastatic liver cancer faces great challenge. From this point of view, finding therapeutic targets for HCC is of great significance for metastatic liver cancer.

Luo et al confirmed that PCBP1-AS1 was abnormally expressed in patients with liver metastases compared to adjacent normal tissues of HCC. It was found that the up-regulation of PCBP1-AS1 promoted HCC proliferation and transfer through function analysis. Interestingly, The physical interaction between PCBP1-AS1 and Polyc-RNA-binding protein 1 (PCBP1-AS1, a multifunctional RNA-binding protein, belongs to the RNA-binding protein subfamily and has specific binding activity to the polycistronic region of RNA and regulate gene expression. In addition, Polyc-RNA-Binding Protein [PCBP1] inhibits the invasion of HCC cells and has been identified as a potential tumor suppressor gene.^[18]^ PCBP1-AS1 and PCBP1 are antisense RNAs for each other, which can complement specific nucleotide sequences of other transcripts and can play different roles in regulating the expression of target genes after transcriptional level by various mechanisms.) antibodies was confirmed by RNA pull-down assay. PCBP1-AS1 and PCBP1 can be detected together in The protein information resource^[19]^. High expression or silencing of PCBP1-AS1 in HCC cancer cells can affect PCBP1 protein levels, and PCBP1-AS1 was negatively correlated with PCBP1 protein levels. Which in turn affect the expression of PCBP1 downstream genes PRL-3 and p -AKT (pT308 and pSer473).^[20]^ When the expression of PCBP1-AS is significantly enhanced, the phosphorylated active forms of PRL-3 and AKT in Hepatocellular carcinoma cellula are increased, and the expression levels of PRL-3 and AKT phosphorylated active forms in HCC cells can be sharply reduced when PCBP1-AS1 is reduced. Therefore, PCBP1-AS1 is positively correlated with the activation of the PRL-3-AKT signaling routing. Modulating Polycytosine binding protein 1 with PRL-3-AKT is consideredas a effective channel to inhibit oncology progress. The study by Luo et al^[21]^ showed that PCBP1-AS1 has a very important role in the development of hepatocellular carcinoma, especially in the proliferation and metastasis mechanisms of cancer cells.

### 2.3. Vulvar squamous cell carcinoma

Vulvar squamous cell carcinoma (VSCC) as a gynecologic tumor^[22]^ is the most common tumor of the vulva.^[23]^ Most of them are primary vulvar squamous cell carcinoma, one of the many malignant tumors of the female reproductive organs, with smaller survival rate in late stages, which poses a great threat to the health of elderly women. Vulvar itching, vulvar nodules, local lumps and ulcers are the main symptoms that greatly affect normal life. Therefore, finding molecular targets that inhibit the development of SCC cells is of considerable significance to prevent further deterioration of VSCC.

Evidence from Wang et al^[24]^ showed a significant decrease in protein expression of PCBP1-AS1 in patients with vulvar squamous cell carcinoma. The relative expression levels of PCBP1-AS1 and tumor necrosis factor receptor-associated factor (TRAF)-5 mRNA in cancerous tissues were determined according to q RT-PCR technology, and a certain clinic-pathological correlation was found between the 2. In turn, the activation of the NF-κB/p65 pathway and NF-κ B/c-Re l pathway mediated by TRAF5 in normal vulvar tissues and the expression of TRAF5 protein in corresponding cancer tissues decreased accordingly. It was also found that the up-regulation of PCBP1-AS1 played a role in tumor inhibition proliferation and promotion of apoptosis in the progress of VSCC. In addition, when Polycytosine binding protein 1 antisense expression is up-regulated, TRAF5 mRNA increases, while TRAF5 expression cannot affect the expression of Polycytosine binding protein 1 antisense after up-regulation and down-regulation. However, up-regulation of TRAF5 expression can increase the activity of NF-k B, and down-regulation of TRAF5 can reduce the activity of NF-κ B.^[20]^ In conclusion, NF-κ B is positively correlated with PCBP1-AS1 expression. From this point of view, TRAF5 protein acts as a link between PCBP1-AS1 and NF-κ B pathway, and jointly affects the proliferation and apoptosis of VSCC SW954 cells. Therefore, Polycytosine binding protein 1 antisense may act as an tumor suppressor gene to influence the development of vulvar squamous cell carcinoma and provide new ideas for the treatment of vulvar squamous cell carcinoma.

### 2.4. Hodgkin lymphoma

Hodgkin lymphoma (HL) is a B-cell lymphoma, a rare malignant tumor.^[25]^ It is one of the first systemic tumors to be proven curable by radiotherapy and multi-agent chemotherapy, but recurrence after treatment is also a major treatment challenge due to the extensive, nonspecific manifestations of the disease and the unclear mechanism of disease occurrence and progression. Studies identified 18 LncRNAs dysregulate between early recurrence and late recurrence of this tumor. Competing endogenous (CeRNA) network includes 6 LncRNAs, 116 mRNA and 121 miRNAs. One of the most critical LncRNAs for HL recurrence is PCBP1-AS1. The down-regulation of PCBP1-AS1 is the main expression gene of HL relapse. Interestingly, it was found that Polycytosine binding protein 1 antisense 1 is one of the crux regulators in this electric network, and PCBP1-AS1 can competitively bind to multiple miRNA axes and mRNA. In short, PCBP1-AS1 can regulate mRNA through competitive binding. These have been confirmed in the ceRNA network.^[26]^ However, the specific results still need to be confirmed in larger clinical trials. In short, PCBP1-AS1 is down-regulated in HL relapse progression, and it is believed that this study will provide new ideafor potential therapeutic and prognostic targets for HL.

### 2.5. Lung adenocarcinoma

Lung adenocarcinoma (LUAD), as the world’s leading cancer killer, is the commonest type of lung tumor.^[27]^ Patients with Lung adenocarcinoma have extremely low survival rates.^[28]^ Research confirms that rising incidence is the biggest cause of cancer-related death worldwide. LncRNA has been proved to play an vital role in the occurrence and progression of lung adenocarcinoma.^[29]^ Li research proved that down-regulation of PCBP1-AS1 significantly enhanced the migration and invasion of cancer cells.^[30]^ On the contrary, up-regulation of PCBP1-AS1 could inhibit the metastasis of cancer cellula. In order to explore the correlation between PCBP1-AS1 expression and TME for lung adenocarcinoma. Li team used CIBERSORT algorithm (A method for assessing tissue cell composition from gene expression profiles) to study the level of tumor-initiating cells subpopulation,^[31]^ and obtained 21 types of immune cell profiles in Lung adenocarcinoma patients. Through observation, it was found that the level of monocytes, neutrophils and activated NK cells in LAUD samples was abnormal. In addition to this, a significant correlation was found between PCBP1-AS1 expression and a range of killer and regulatory cells. Such as stimulated DCs, resting DCs, mast cells, eosinophils, and abnormal T cell regulation. These correlations are sufficient to confirm the relationship between PCBP1-AS1 expression and the immune microenvironment. In addition, inhibition of PCBP1-AS1 induced decreased E-cadherin expression and increased expression of N-cadherin, vimentin and Snail expression. Thus, the study was hypothesized that PCBP1-AS1 targeting the epithelial-mesenchymal transition pathway inhibited the metastasis of LUAD. However, we have not yet found more relevant literature and hope that more mature clinical studies in the future will support the potential use of PCBP1-AS1 as a prognostic biomarker and potential therapeutic target in the future treatment of LUAD.

### 2.6. CC

CC, abbreviated as CC, is one of the most common malignancies of the female reproductive system, with a high mortality rate and an increasing prevalence among young women. Two thousand twenty one study shows CC takes the 4th place in cancer incidence and mortality among women.^[32,33]^ Although diagnostic and treatment strategies have been developed, the cancer is characterized by easy metastasis and recurrence, resulting in a poor prognosis for patients with CC. Therefore, the discovery of biomarkers associated with CC and specific therapeutic targets is an urgent task.

A series of researches have proved that PCBP1-AS1 is took part in the evolution of carcinoma of uterine cervix. Carcinoma of uterine cervix cells were significantly up-regulated compared with normal cervical cells compared with PCBP1-AS1. The main significant pathways of PCBP1-AS1 include p53^[34]^ signaling pathway and Notch signaling pathway,^[35]^ but not only that, it has been shown that there are also other genes that can inhibit and activate p53 signaling and Notch signaling pathways to promote proliferation and inhibit apoptosis of CC cells. In addition, the expression of PCBP1-AS1 is strongly correlated with the level of immune infiltration. When PCBP1-AS1 is overexpressed, the level of M2 macrophages and activated NK cells decreases, the important biological barrier residing in the cervix is destroyed, and the ability to quickly recognize and kill viruses is weakened. Experiments have also shown that PCBP1-AS1 down-regulation can inhibit the proliferation, invasion and migration of cancer cells.^[36]^ Based on this study, it is expected that PCBP1-AS1 will be used as a de novo marker of CC to explain the immune environment promoting the development of CC.

### 2.7. Glioma

Gliomas are characterized by high recurrence rates, high mortality, and low cure rates.^[37]^ Besides, uncontrolled proliferation, cell heterogeneity and diffusion.^[38]^ As a result, the prognosis for patients with high-grade gliomas is very poor. Exploring new molecular biomarkers related to glioma pathogenesis is a great undertaking. Researches showed that the progress and prognosis of glioma are strongly correlated to abnormal methylation of some LncRNA promoter regions.^[39]^ Among them, researches have demonstrated that PCBP1-AS1 is involved in the malignant progression of glioma cell. Loss-of-function assays confirmed that knock-down PCBP1-AS1 inhibited the propagation, invasion and transfer of tumor cells. Meng et al analyzed the expression level of Polycytosine binding protein 1 antisense1 in this tumor cell by RT-PCR, and the results showed that PCBP1-AS1 was abnormally high expressed in LN18 and T98G. In addition, its location in glioma cells was analyzed. Polycytosine binding protein 1 antisense1 was expressed in both the nucleus and cytoplasm by nuclear cytoplasmic isolation. Therefore, by silencing the expression of PCBP1-AS1 in glioma cells, the clonal formation ability of LN18 and T98G cells was significantly reduced, reducing the proliferation, invasion and migration of glioma cells.^[40]^ Meantime, a key role of Inc RNA promoter region methylation in gliomas has been highlighted, but the specific factors that mediate methylation of the LncRNA promoter region have not been well studied. Therefore, PCBP1-AS1 may become a prognostic biomarker and potential target for the treatment of glioma patients.

### 2.8. Colorectal cancer

Colorectal cancer is referred to as colorectal cancer (CRC), which is the third most common cancer in China. It is also the second leading cause of cancer-related death.^[41]^ Late treatment of colon cancer still faces great challenges, and dysregulation of cellular metabolism has become a key hallmark of cancer.^[42]^ Numerous research evidence suggests that mRNA and long noncoding RNAs play an significant role in the evolution of CRC.^[43]^ The study found that PCBP1-AS1 up-regulation can isolate several mi RNAs such as has-miR-582-5p and has-miR-198. It also promotes the proliferation, invasion and drug resistance of colorectal cancer cells. In addition, studies have shown that hsa-miR-1827 may take part in adjusting the aggressiveness and resistance of colorectal cancer cellula. Isolation of miR-198 increased the expression of FUT8 and proliferation and invasion of CRC. In addition, to sum up, the analysis of the interaction network of LncRNA-mi RNA-mRNA based on genetic engineering can offer new model of thinking for the early therapy of colorectal cancer.^[44]^

In addition, PCBP1-AS1 may take part in the progression of squamous cell carcinoma in a small number of literature, but there is little research on its specific regulatory mechanism. However, more research is needed to demonstrate the potential use of PCBP1-AS1 as an OSCC biomarker or therapeutic target.

## 3. Regulatory mechanism of LncRNA

In summary, PCBP1-AS1 acts as an oncogenic factor in CRPC, HCC, CC, glioma and CRC. However, act as tumor suppressors in VSCC, LUAD and HL. The table below summarizes the involvement of PCBP1-AS1 in the evolution and progression of a variety of different cancers through various regulatory mechanisms (Table 1). Although also acting as oncogenes or oncogenes, their regulatory pathways are each characterized by:

**Table 1:** Tumor-associated LncRNA PCBP1-AS1 reported biological functions and the affected pathways.

Cancer types	Biological significance	Genes/proteins/pathways	Attribute	Expression	Refs.
Castration-Resistant prostate cancer	Proliferation, migration	PCBP1-AS1/USP22-AR/AR-V7	Oncogene	Up-regulation	^[15]^
Hepatocellular carcinoma	Proliferation, migration	PCBP1-AS1/PRL-3-AKT/PCBP1	Oncogene	Up-regulation	^[20,21]^
Vulvar squamous cell carcinoma	Inhibitor of proliferation, enhance apoptosis	PCBP1-AS1/TRAF5/NF-kB	Inhibitor	Down-regulation	^[24]^
Hodgkin lymphoma	Proliferation	PCBP1-AS1/mRNA	Unknown	Down-regulation	^[26]^
Lung adenocarcinoma	Inhibitor of migration	PCBP1-AS1/EMT	Inhibitor	Down-regulation	^[29–31]^
Cervical cancer	Invasion, proliferation, migration	PCBP1-AS1/p53/notch	Oncogene	Up-regulation	^[34–36]^
Glioma	Proliferation, migration and invasion	Unknown	Oncogene	Up-regulation	^[40]^
Colorectal cancer	Inhibitor, proliferation, tolerance	LncRNA-miRNA-mRNA	Unknown	Up-regulation	^[44]^

AR = androgen receptor, LncRNA = long noncoding RNA, miRNA = microRNA, mRNA = messenger RNA, PCBP1 = Polyc-RNA-Binding Protein, PCBP1-AS1 = polycytosine binding protein 1 antisense1, TRAF = tumor necrosis factor receptor-associated factor, USP22 = ubiquitin-specific peptidase 22.

### 3.1. Interaction with proteins

LncRNA-protein interactions are a key aspect of many cellular processes.^[45]^ Several databases such as CLIPZ,^[26]^ StarBase,^[46]^ doRiNA^[47]^ and NPInter^[48]^ provide public data analysis of protein - LncRNA interactions. PCBP1-AS1 can bind and interact with AR proteins., During CRPC, up-regulation could first promote the binding of PCBP1-AS1 to ubiquitin-specific peptidase 22 (USP22) and the NTD structural domain of AR/AR-V7, and subsequently promote the stability of the AR/AR-V7 complex, inhibit AR ubiquitination, and promote the resistance of cancer cells to enzalutamide during CRPC treatment. USP22 belong to the family of USPs, which are involved in the regulation of tumor cell cycle-related genes and are closely associated with malignant cell proliferation. According to the study, USP14^[49]^, USP22^[50]^ and USP26^[51]^ were immunoprecipitated and only USP22 could bind to PCBP1-AS1.

Besides, PCBP1-AS1 can PCBP1-AS1 physically interacts with PCBP1 protein^[20]^ (The PCBP1 gene locus is located at 2p12-13 and has 3 kH domains). PCBP1-AS1 was positively correlated with PRL-3 protein expression, which in turn promoted AKT phosphorylation levels at pS473 and pT308, ultimately affecting the AKT pathway. High expression activates the PRL-3-AKT signaling pathway and opposes the expression of PCBP1 (PRL-3 and p-AKT are the downstream genes of PCBP1). PCBP1-AS1 induces the activation of this signaling pathway through high expression, thereby regulating the PCBP1-PRL-3-AKT axis to promote HCC proliferation and metastasis. TRAF is an intracellular signal transduction protein that binds to the cytoplasmic region of members of the tumor necrosis factor receptor superfamily and is involved in the activation of multiple signaling pathways. This family includes TRAF1-6, of which TRAF5 plays an important role in VSCC. PCBP1-AS1 promotes TRAF954 mRNA expression in SW5 cells to indirectly regulate NF-κB pathway proteins (including p65, p-p65, c-Rel and IκBα proteins) pathway activity.^[52]^ Up-regulation of PCBP1-AS1 inhibits VSCC SW954 cell proliferation, migration and invasion.

PCBP1-AS1 inhibition induced decreased E-calmodulin expression and increased N-calmodulin, Vimentin and snail expression, and PCBP1-AS1 inhibited LUAD metastasis by targeting the epithelial-mesenchymal transition pathway. p53 plays a complex role not only in promoting cell cycle, cellular senescence and apoptosis, but also has important regulatory roles in proliferation, invasion and metastasis of CC.^[53,54]^

### 3.2. Interaction with RNA

In addition to interacting with proteins, PCBP1-AS1 can also interact with RNA to participate in cancer regulation. In recent years, the LncRNA-miRNA-mRNA network and ceRNA network have been of great importance for the potential analysis of LncRNA, in which PCBP1-AS1 acts as a key regulator. In HL PCBP1-AS1 can regulate more than 60 mRNAs, has-miR-34-5p, has-miR-9c-5p, has-miR-34-5p, has-miR-539-5p, has-miR-122-5p, has-miR-124a, has-miR-3b-449p, has-miR-449-5p and has-miR-491-5p through competitive binding to participate in the immune regulatory system. The ceRNA hypothesis has important mechanisms that explain how LncRNAs can regulate protein-coding genes and regulate disease progression after competitive binding to miRNAs.^[55]^ Dysregulation of LncRNAs such as PCBP1-AS1, UCA1 and SNHG16 in CRC can isolate several miRNAs such as has-miR-582-5p and has-miR-198 and promote proliferation, invasion and drug resistance in Colorectal Cancer cells.

### 3.3. Methylation of LncRNA promoter region

DNA methylation as an important epigenetic modification can maintain gene integrity and regulate gene expression.^[56]^ In recent years, LncRNA methylation as a new player can also bind directly to transcription factors or to miRNAs in the ceRNA network, which can also function as epigenetic modifiers in tumors.^[57,58]^ According to the Cancer Gene Atlas database, 9 related LncRNA methylation genes were initially identified, and it was found that down-regulation of PCBP1-AS1 expression could reduce the clonogenic ability of LN18 and T98G cells, which in turn led to a significant decrease in the migration and invasive ability of glioma cells.

So far, some related researchers have also indicated that there are also many limitations in the studies on PCBP1-AS1. In addition, there is a limited amount of highly relevant literature in PubMed. Studies do demonstrate some correlation, but functional studies have not yet been elucidated, leading to a summary of certain mechanisms that still seem to be ambiguous.

## 4. Conclusion

**Figure 1.**
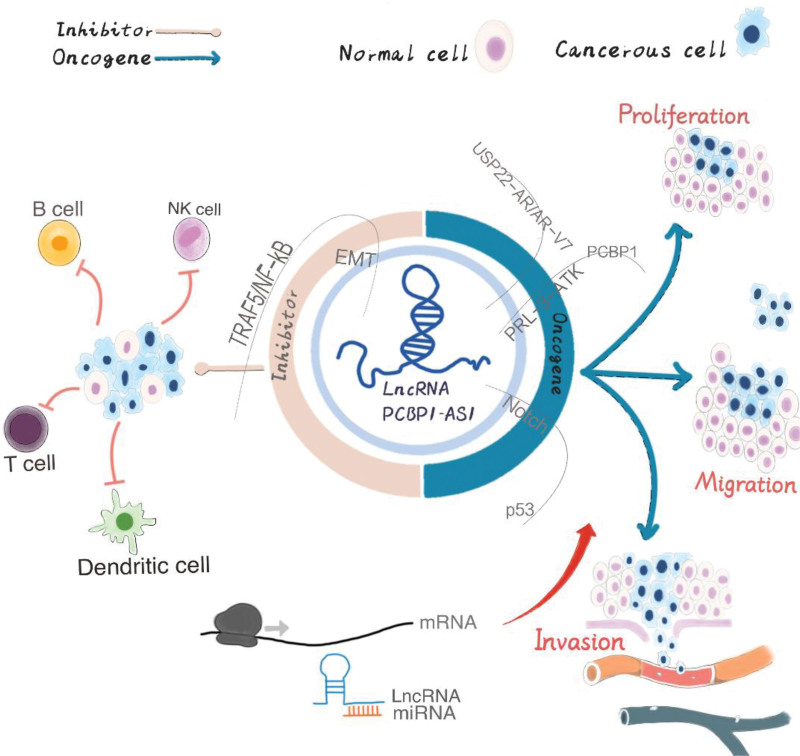
Mechanism of action of PCBP1-AS1 and its effect on tumor development. PCBP1-AS1 = polycytosine binding protein 1 antisense1.

In conclusion, PCBP1-AS1 can regulate the occurrence and development of a series of cancer for instance prostate cancer, lung adenocarcinoma, hepatocellular carcinoma, CC, vulvar squamous cell carcinoma, Hodgkin lymphoma, glioma, colorectal cancer and oral squamous cell carcinoma. PCBP1-AS1 is involved in many biological processes such as propagation, invasion, transfer, metastasis and apoptosis of tumor cells, and drug resistance. Figure 1 shows the regulation of tumor development by PCBP1-AS1. However, malignant tumorigenesis is the result of multiple factors, and LncRNA PCBP1-AS1, as a newly discovered biomarker in recent years, is involved in the regulatory process of numerous tumorigenesis and development described above, which may provide a new vision and direction for cancer treatment to some extent.

## Author contributions

**Supervision:** Wenfei Zheng.

**Visualization:** Wenfei Zheng, Yanping Wu.

**Writing – original draft:** Yanping Wu, Jie Mou, Yuling Liu, Wenfei Zheng.

**Writing – review & editing:** Wenfei Zheng.
